# Supplementary material for: G-Banding and Molecular Cytogenetics Detect Novel Translocations and Cryptic Aberrations in Human Immortal Endothelial Cells
Source: Int J Mol Sci. 2024 Jul 20;25(14):7941. doi: 10.3390/ijms25147941 (PMC11276908; doi:10.3390/ijms25147941)
Supplement: Supplementary file 1 [file ijms-25-07941-s001.zip › ijms-3083186-supplementary.pptx]

## Slide 1
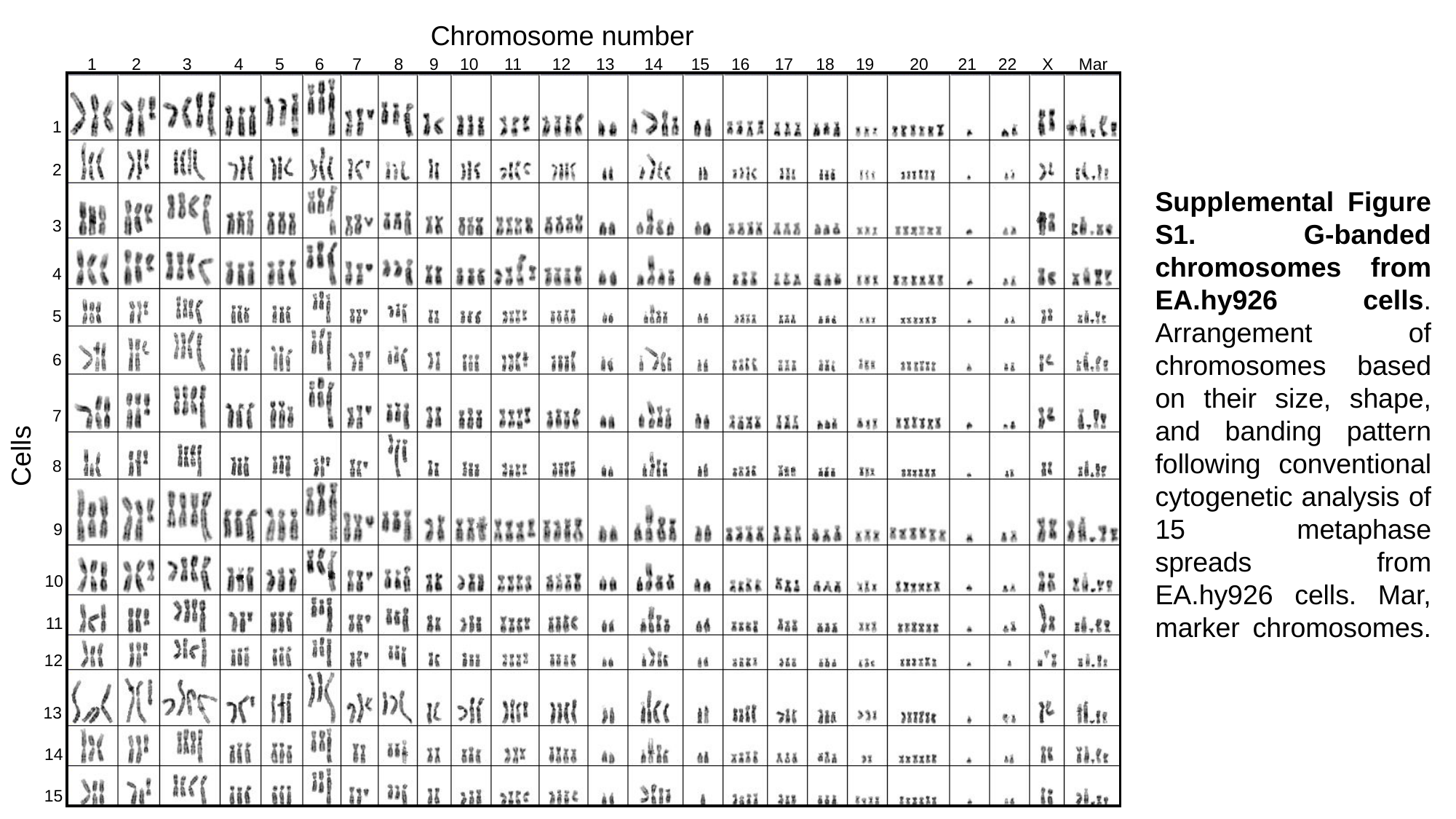

Chromosome number
1
2
3
4
5
6
7
8
9
10
11
12
13
14
15
16
17
18
19
20
21
22
X
Mar
1
2
Supplemental Figure S1. G-banded chromosomes from EA.hy926 cells. Arrangement of chromosomes based on their size, shape, and banding pattern following conventional cytogenetic analysis of 15 metaphase spreads from EA.hy926 cells. Mar, marker chromosomes.
3
4
5
6
7
Cells
8
9
10
11
12
13
14
15

## Slide 2
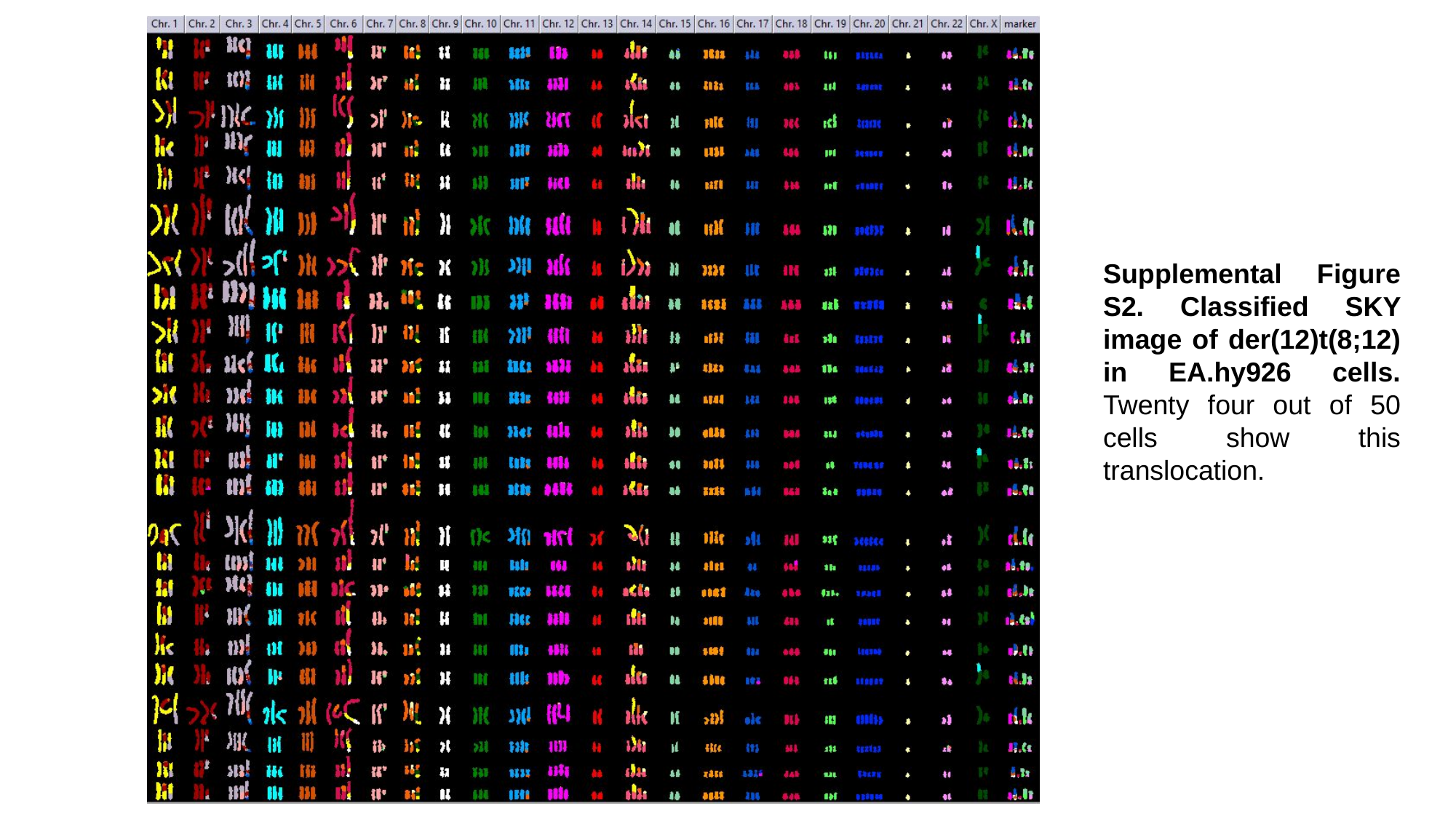

Supplemental Figure S2. Classified SKY image of der(12)t(8;12) in EA.hy926 cells. Twenty four out of 50 cells show this translocation.

## Slide 3
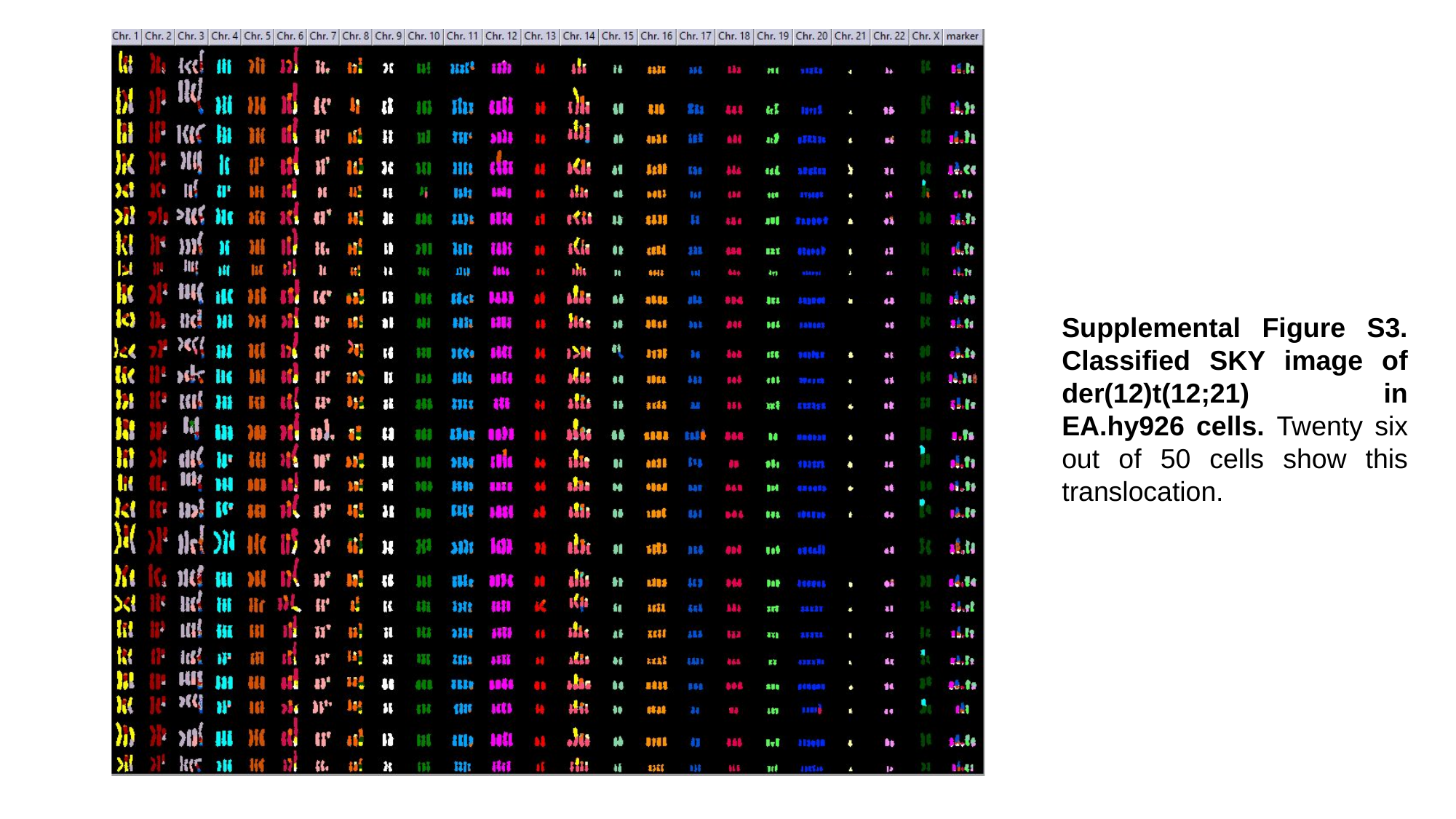

Supplemental Figure S3. Classified SKY image of der(12)t(12;21) in EA.hy926 cells. Twenty six out of 50 cells show this translocation.

## Slide 4
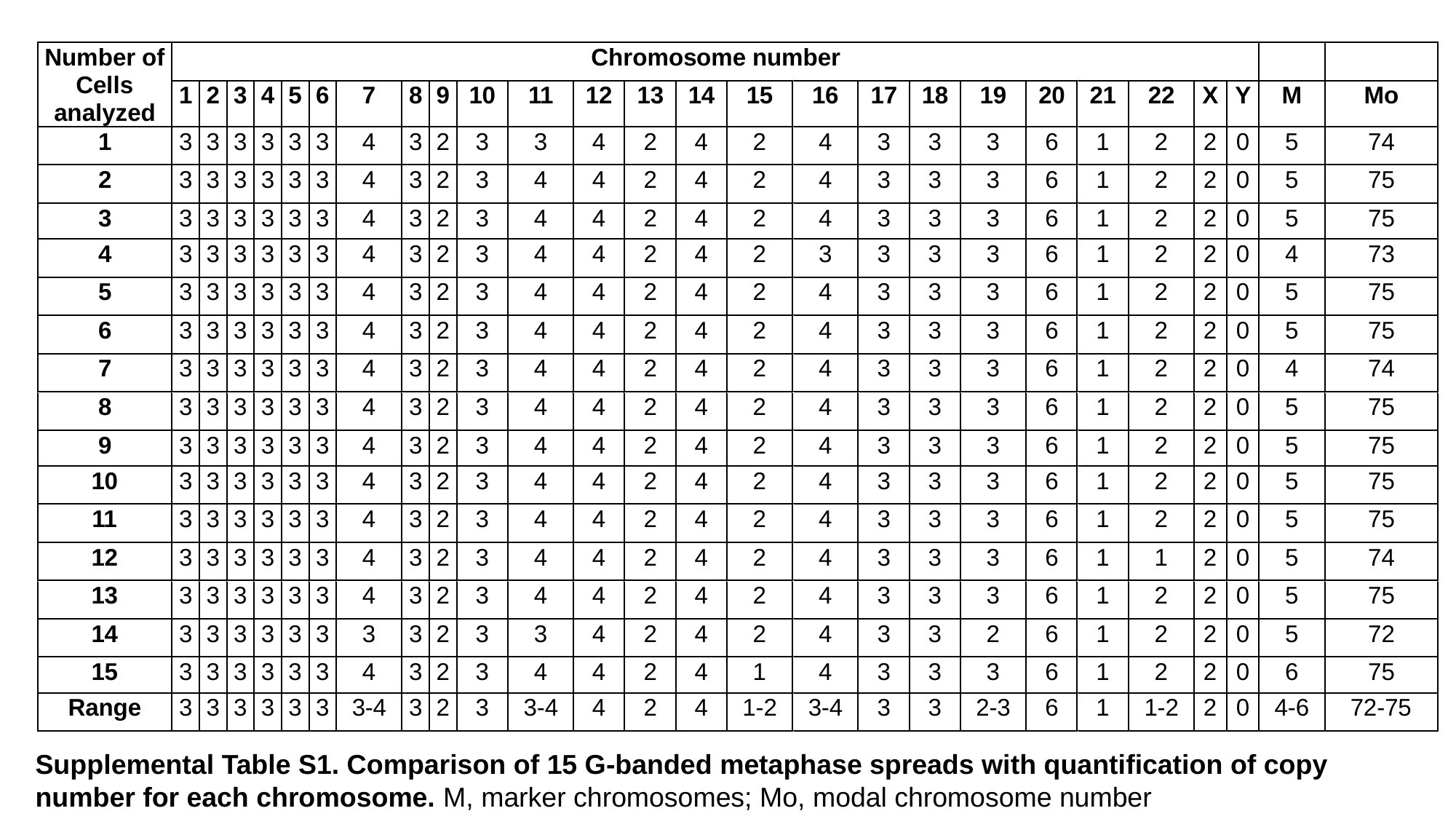

Supplemental Table S1. Comparison of 15 G-banded metaphase spreads with quantification of copy number for each chromosome. M, marker chromosomes; Mo, modal chromosome number
